# Biological Effects of a Fine Fiber Film Treated With a Lotion to Improve Dry Skin

**DOI:** 10.1111/srt.70161

**Published:** 2025-05-05

**Authors:** Shun Sasaoka, Yu Gabe, Masayuki Uchiyama, Anita Stepp, Asuka Imai, Akira Hachiya, Akira Kiyomine

**Affiliations:** ^1^ Kao Corporation, Skin Care Products Research Sumida‐ku Tokyo Japan; ^2^ Kao Corporation, Biological Science Research, Kotobuki‐cho Odawara‐shi Kanagawa Japan; ^3^ Kao R&D, Kao USA Inc. Cincinnati Ohio USA

**Keywords:** dry skin, fine fiber, immunohistochemical analysis, lotion, proteome analysis, suprabasin

## Abstract

**Background:**

Dry skin is a universal skin concern that is often accompanied by itching, but moisturizers have not completely solved this issue. We found that the combination of a moisturizer (lotion) and an ultra‐thin film of fine fiber (FF) that we developed improved dry skin early, but the biological mechanism within the skin remained unclear.

**Materials and Methods:**

Eight (Study 1) and seven (Study 2) subjects used a lotion with and without the FF film on their lower legs. In addition to measurement of visual skin dryness scores and conductance, proteomic analysis of stratum corneum (SC) samples at baseline and 14 days after treatment in Study 1 and immunohistochemical staining of skin biopsy samples 3 or 5 days after treatment in Study 2 were performed.

**Results:**

Skin dryness scores and conductance improved more when the lotion was used with the FF film than with the lotion alone. The protein expression level of suprabasin, which is related to epidermal differentiation and barrier function, increased within a few days near the granular layer, as well as in the SC after 14 days. Proteomic analysis using SC samples showed that the FF film treatment with lotion decreased annexin A2, a dry skin marker, whereas it increased retroviral‐like aspartic protease 1, which is related to skin water content, more than lotion alone.

**Conclusion:**

The present findings suggest that lotion with the FF film initiates restoration of epidermal homeostasis, leading to early improvement of dry skin.

## 1 Introduction

Dry skin is a universal skin concern that is often accompanied by itching, which decreases quality of life (QOL) [1, 2, 3]. Dry skin often appears dull, scaly, and flaky, and it may induce an itching, stinging, or burning sensation and redness. Moisturizers (lotions) have long been used for dry skin care, but consumers’ concerns have not yet been exhausted, and moisturizers have not completely solved this issue. We developed fine fiber (FF) technology that can form a film of ultra‐fine fibers on the skin and showed that the combination of a commercial lotion and the FF film improves dry skin faster than a lotion alone. The moisture permeability of the FF film can be controlled by the combination of the FF film and the formulation, and it was also found that there is a combination suitable for improving dry skin [4].

The cause of dry skin is thought to be a dysfunction of processes related to epidermal differentiation and water retention capacity, and the improvement of dry skin symptoms in our study was assumed to be the result of the improvement of such biological mechanisms. However, dermatological insight into the application of lotion with the FF film to dry skin is lacking. Skin biopsy specimens are often analyzed to determine the biological changes that have occurred inside the skin [5]. Although skin biopsy is a useful method for easily and accurately examining processes within the skin, it is extremely invasive. On the other hand, Kim et al. reported that skin tape stripping, which is less invasive, could be an alternative to skin biopsy [6, 7, 8]. In the case of skin tape stripping, the turnover of the stratum corneum (SC), which is estimated to be about 14 days [9], should be considered. Therefore, considering this period, the skin biological mechanisms induced by lotion and the FF film application to dry skin at the early stage were investigated by analyzing skin tape strips of SC before and 14 days after lotion and the FF film application and skin biopsies at the initial (3 or 5 days) of continuous use. In this report, the changes that occur in the skin when lotion and the FF film are applied to dry skin are described, comparing the results with and without the FF film.

## 2 Materials and Methods

### 2.1 Ingredients of Lotion and Fiber

The lotion was an oil‐in‐water cream formulation, based on glycerin, petrolatum, cetearyl alcohol, and pseudo‐ceramide (cetyl PG hydroxyethyl palmitamide); the full ingredient list is shown in Table S1. The fiber composition for electro‐spinning is shown in Table S2. In our previous study [4], the moisture permeability of various films was evaluated according to the Japanese Industrial Standard Z0208. The moisture permeability of the FF film with lotion and that of the FF film alone used for occlusive treatment were 1489 and 2618 g/m^2^/24 h, respectively. The fiber network on the skin surface was prepared using an electro‐spinning method, as described in the following section.

### 2.2 FF Film on the Skin Surface

To form a thin FF film on the skin surface, the direct‐electrospinning (D‐ES) method was selected, and a device that can provide a uniform, thin, and flexible membrane on the skin was developed. The D‐ES method is a technique in which a positively charged polymer solution is ejected toward the surface of a negatively charged object. The device uses an electric field to control the formation and deposition of the polymer, which consists of a single continuous filament, in an extremely efficient and rapid manner. The polymer solution is ejected from the nozzle tip of the device and flies toward the object surface, with the solvent evaporating as it is attracted by the electric field.

### 2.3 Clinical Studies

These studies consisted of a dry‐out phase (3 days) and a treatment phase (14 days for tape stripping and 3 or 5 days for skin biopsy). During the treatment phase, the lotion was applied to both lower legs, and then the FF film was applied to either the left or the right side. Each subject applied the assigned test product to their lower legs by themselves every day during the treatment phase, ensuring that the lotion was evenly distributed and fully absorbed. Subsequently, the FF solution was applied using a portable device (0.10 mL/min) over the area where the lotion had already been applied until the area was completely and evenly covered (60 s). The FF film remained on the skin surface for at least 16 h until the subject intentionally removed the products, after which the lotion and the FF film were reapplied each day throughout the treatment phase. Eight and seven healthy subjects (Caucasian or African‐American females) whose observer dryness score on each lower leg area was above 2.5 were enrolled in the tape‐stripping study (38–54 years old, clinical study 1) and the biopsy study (38–54 years old, clinical study 2), respectively. Skin measurements were carried out at baseline, Days 7 and 14 for the tape‐stripping study and at baseline and Days 3 or 5 for the biopsy study. Tape stripping samples were obtained at baseline and Day 14. Skin biopsy samples were procured at North Cliff Consultants, Inc. (Cincinnati, OH, USA). The tape stripping study was conducted according to the principles of the Declaration of Helsinki using protocols ethically reviewed and approved by the organization Kao USA Inc. The biopsy studies performed in the United States were approved by Schulman Associates Institutional Review Board, Inc. (Cincinnati, OH, USA) (protocol numbers 17‐124‐NCC/20‐1703, 18‐009‐NCC). Written informed consent to participate in these studies was obtained from each subject before the start of the study.

### 2.4 Skin Condition Measurements

All evaluations were conducted in an air‐conditioned room at 21 ± 1°C and less than 40% relative humidity. Each subject washed both lower legs with a detergent, entered the room, and acclimated for at least 15 min. Then, 5 × 5 cm^2^ areas were marked on the outer surface of each leg where the skin dryness was similar. Skin hydration was measured using a Dermalab Moisture Meter (Cortex Technology, Hadsund, Denmark). Observer dryness scores on each lower leg area were assessed by trained experts according to a reference consisting of 5 grades (0, no dryness; 1, slight flaking; 2, moderate flaking/scaling; 3, marked scaling/slight fissuring; and 4, severe scaling/fissuring) from 0 to 4 with halfway scores (Figure S1).

### 2.5 Proteomic Analysis

SC samples were collected from eight subjects at baseline and Day 14 using a film masking tape (Teraoka Seisakusho, Tokyo, Japan) and then stored in a freezer at −80°C. Tapes were cut into pieces and dipped in 1 mL lysis buffer [10] (7 mol/L urea and 2 mol/L thiourea, 12 mmol/L sodium deoxycholate, 12 mmol/L sodium N‐lauroyl sarcosinate, 100 mmol/L Tris‐HCl; Sigma‐Aldrich, Deisenhofen, Germany). This solution containing tapes was sonicated for 20 min. Dithiothreitol (FUJIFILM, Tokyo, Japan) was added to this solution with a final concentration of 0.1 mmol/L; it was then shaken gently at 37°C overnight. Iodoacetamide (FUJIFILM) was added with a final concentration of 0.5 mmol/L at 25°C for 30 min. The extracted protein concentration was measured using the EZQ protein quantification kit (Thermo Fisher Scientific, Pleasanton, CA, USA) according to the manufacturer's instructions. Furthermore, 2 mL of 50 mmol/L ammonium bicarbonate (FUJIFILM) was added to the sample solutions. Lys‐C protease (FUJIFILM) was added to the sample solutions and incubated for 3 h at 37°C. Subsequently, trypsin protease (FUJIFILM) was added and incubated at 37°C overnight. The sample solutions were divided equally into three tubes, and then 1 mL of ethyl acetate was added, after which trifluoroacetic acid (both FUJIFILM) at a final concentration of 0.5% (v/v) was added to stop the protease reaction. The solutions were shaken for 2 min and centrifuged at 20 000 *g* for 5 min. The lower aqueous layer was collected and dried using an evaporator. The dried residues were dissolved in 0.1% trifluoroacetic acid containing 5% acetonitrile and then purified by solid phase extraction with styrene divinyl benzene disks (SUPELCO, Bellefonte, PA, USA). The purified peptide solutions were dried using an evaporator and finally dissolved in 0.1% formic acid containing 2% acetonitrile (FUJIFILM). Purified peptides were analyzed using nano‐liquid chromatography (LC, Waters nanoAcquity UPLC) coupled with mass spectrometry (MS, Thermo Scientific Orbitrap Velos) and a BEH nanoACQUITY 0.1 mm I.D. × 100 mm column (Waters). In this nanoLC system, a nanoAcquity binary pump was connected to two mobile phases (A, 0.1% formic acid containing water; B, 0.1% formic acid containing 80% acetonitrile‐water) with a flow rate of 500 nL/min. The mobile phases were consecutively programmed as follows: an isocratic elution of A 95% (B 5%) between 0 and 5 min, a linear gradient of A 95%–50% (B 5%–50%) between 5 and 125 min, an isocratic elution of A 5% (B 95%) for 25 min, and an isocratic elution of A 95% (B 5%) between 150 and 180 min to re‐equilibrate the column (a total runtime of 180 min). Parameters for mass spectrometry were as follows: spray voltage, 1800 V; capillary temperature, 250°C; mass‐to‐charge ratio (*m/z*) range for full scan, 300–1250; resolution for full scan, 60 000 at *m/z* 400; fragmentation method, collision‐induced dissociation (CID), data‐dependent acquisition, top 15 precursor ions; exclusion time, 180 s; *m/z* range for MS2, 100–2000; and collision energy, 35 eV.

### 2.6 Immunohistochemical Analysis

Biopsied skin samples were embedded in optimal cutting temperature (OCT) compound. Frozen skin sections were fixed with cold acetone. Tissues were then incubated in 1% bovine serum albumin (BSA)/PBS, followed by treatment with rabbit anti‐suprabasin antibody (Novus Biologicals, Littleton, CO, USA; 1:500) or rabbit anti‐bleomycin hydrolase antibody (Atlas Antibodies, Bromma, Sweden; 1:200). Incubation was performed with Alexa‐labeled secondary antibodies (Life Technologies, Carlsbad, CA, USA; 1:500) corresponding to the primary antibody, followed by nuclear staining with 4’6‐diamidino‐2‐phenylindole (DAPI) in a mounting solution (Vector, Burlingame, CA, USA). Images were obtained with a Zeiss LSM710 Confocal Microscope (Carl Zeiss Microscopy GmbH, Jena, Germany). The immunofluorescence intensity of these images was measured and quantified using Image J.

### 2.7 Proteomics Data Analysis

The raw mass spectrometry data were processed using mzR [11], a Bioconductor package. The processed MS/MS spectra were searched using rTANDEM [12], a Bioconductor package against the UniProtKB human reference proteome peptide database. Parameters for the database search with rTANDEM were as follows: cleavage site, cleavage C‐terminal to every lysine or arginine, except when accompanied by a proline; potential modification of methionine oxidation; static change of cysteine carbamidomethylation; maximum miss cleavage is up to one; precursor mass tolerance, 10 ppm; fragment mass tolerance, 0.8 Da; and peptide spectral matches (PSMs) were validated using a target‐decoy search [13] at a 1% false discovery rate (FDR). To evaluate the protein levels, the protein amount index (PAI) was defined as follows. The peptide peak intensity (PPI) was defined as the sum of the peak intensities of all peptides used for protein identification. In this study, K1C10 (Keratin, type I cytoskeletal 10), which showed the highest PPI, was defined as the standard protein, and the ratio of the PPI of K1C10 in each sample to the maximum PPI of K1C10 in all samples was defined as the weight (wt). The PPI of the protein in each sample was normalized by wt, and the log10‐transformed value was defined as the amount of protein. Each protein function was assigned using Uniprot REST API with UniProt.ws [14], a Bioconductor package.

PPI=∑peptidepeakintensity

$$\;wt_{i}=\frac{PPI_{i}\left(\mathrm{standard}\;\mathrm{protein}\right)}{PPI_{k}\left(\mathrm{standard}\;\mathrm{protein}\right)}$$

$$PAI_{\left(\mathrm{each}\;\mathrm{protein}\right)}=\log_{10}\frac{PPI_{\left(\mathrm{each}\;\mathrm{protein}\right)}}{wt_{i}}$$

i: i‐th sample, k: the sample that had the maximum PPI of the standard protein

### 2.8 Statistical Analysis

In clinical study 1, observer scores and conductance changes were evaluated using the paired Wilcoxon signed‐rank test (with FDR correction), and differences of *p* < 0.05 were considered significant. Fold changes of each SC protein between baseline and day 14 were assessed by a paired Wilcoxon signed‐rank test (with FDR correction). The calculated *p* values from paired Wilcoxon signed‐rank tests were converted to *q*‐values according to the Storey method [15], using the FDR threshold of 5%. These values can be regarded as adjusted *p* values, and differences of *q* < 0.05 were considered significant for each protein level. In clinical study 2, changes in immunofluorescence intensities were evaluated using the paired *t*‐test.

## 3 Results

### 3.1 Clinical Study 1

**FIGURE 1:**
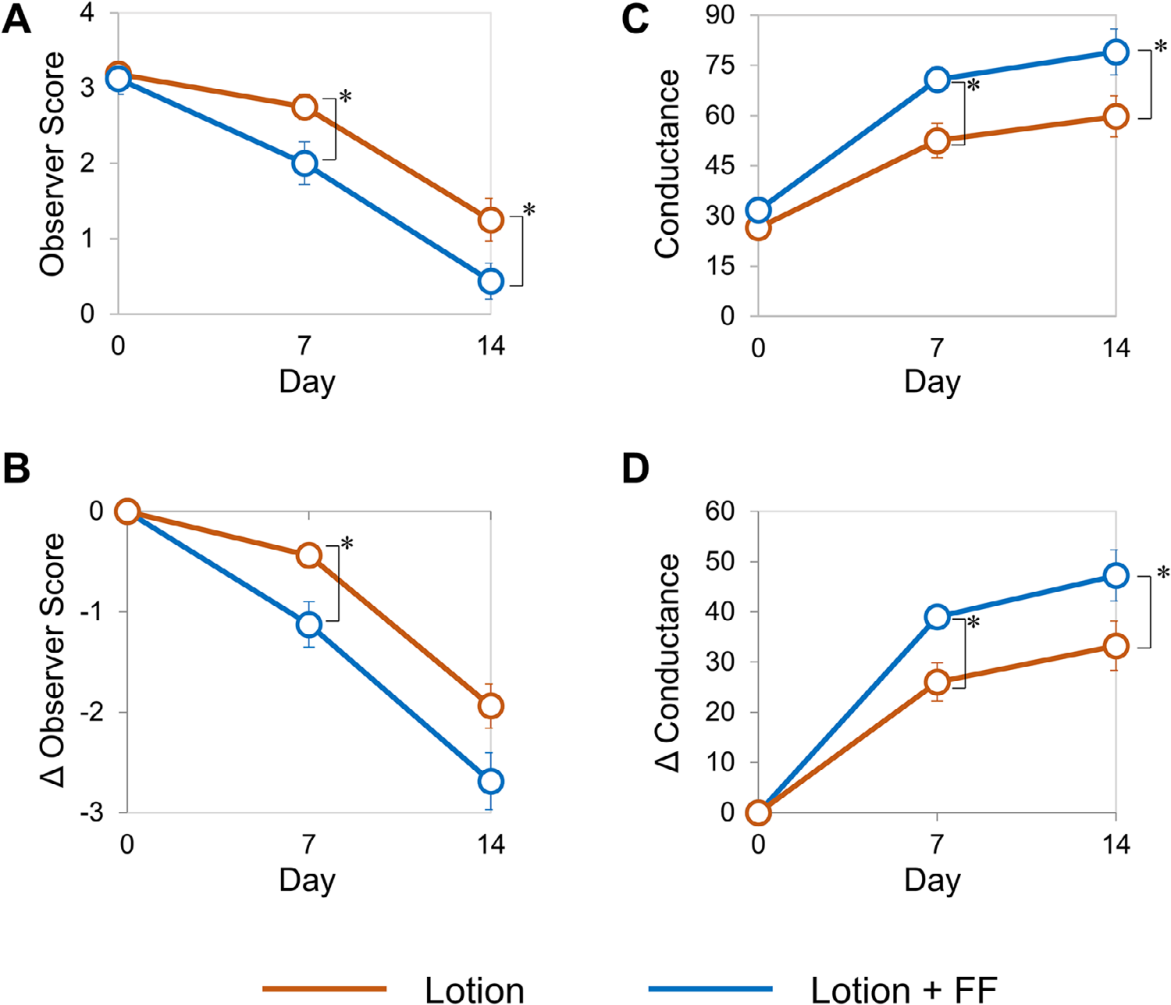
Serial changes in skin dryness and hydration after the different treatments in clinical study 1. (A) The observer score of skin dryness. (B) Changes in the observer score of skin dryness from Day 0. (C) Skin conductance values. (D) Changes in skin conductance values from Day 0. Values are expressed as means ± SE (*n* = 8). The paired Wilcoxon signed‐rank test (with FDR correction) was used for statistical analysis. *: *p* < 0.05.

In a previous study [4], we compared lotion alone and lotion with the FF film in 17 healthy female subjects (initial dryness score 1–4) with dry skin on the lower legs. The results showed that the combination of the FF film improved observer dryness scores and conductance in the first week more than lotion alone. In the present study, lotion alone and with the FF film were compared in 8 of the above 17 patients with high initial observer dryness scores (initial score ≥ 2.5) to investigate the biological changes in the skin that contributed to this demonstrated improvement. Initial observer scores were 3.2 ± 0.1 and 3.1 ± 0.2 (mean ± standard error [SE]) for lotion alone and with the FF film, respectively, with scores significantly lower with the FF film at both 1 and 2 weeks than without it (Figure 1A). The observer scores decreased during 2 weeks of continuous use with and without the FF film, and a significant decrease was observed after 1 week of continuous use with the FF film (Figure 1B). The conductance values were significantly higher with the FF film than without the FF film at both 1 and 2 weeks (Figure 1C). In addition, conductance at 1 week with the FF film was higher than at 2 weeks without the FF film. The conductance increased with and without the FF film during continuous use, and a significant increase was observed after 1 and 2 weeks of continuous use with the FF film (Figure 1D).

**FIGURE 2:**
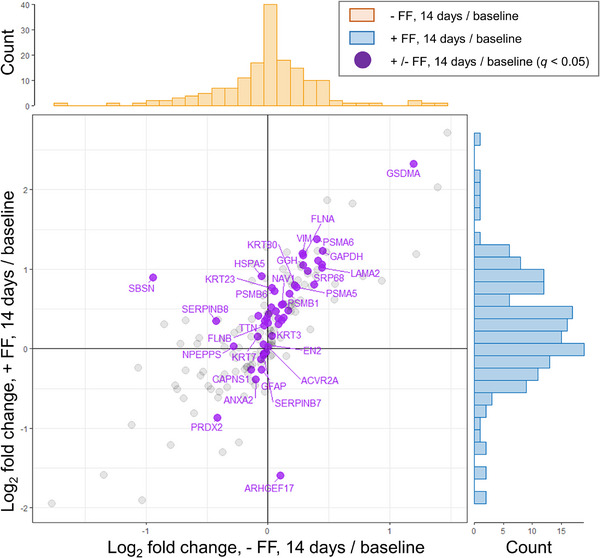
SC proteome after 14 days showing enhanced or reduced levels. A scatter plot showing the log_2_ fold change in protein levels after treatment. Purple dots in the scatter plot indicate all significantly changed proteins between with and without FF (paired Wilcoxon signed‐rank test with FDR correction, *q* < 0.05). The marginal distributions of the scatter plot are shown as histograms at the sides of the plot (orange: without FF, blue: with FF). The histograms represent the frequency distribution of log_2_ fold changes of SC proteins. The x‐axis of the histograms shares the same log_2_ fold change scale as the scatter plot, and the y‐axis represents the protein counts.

**FIGURE 3:**
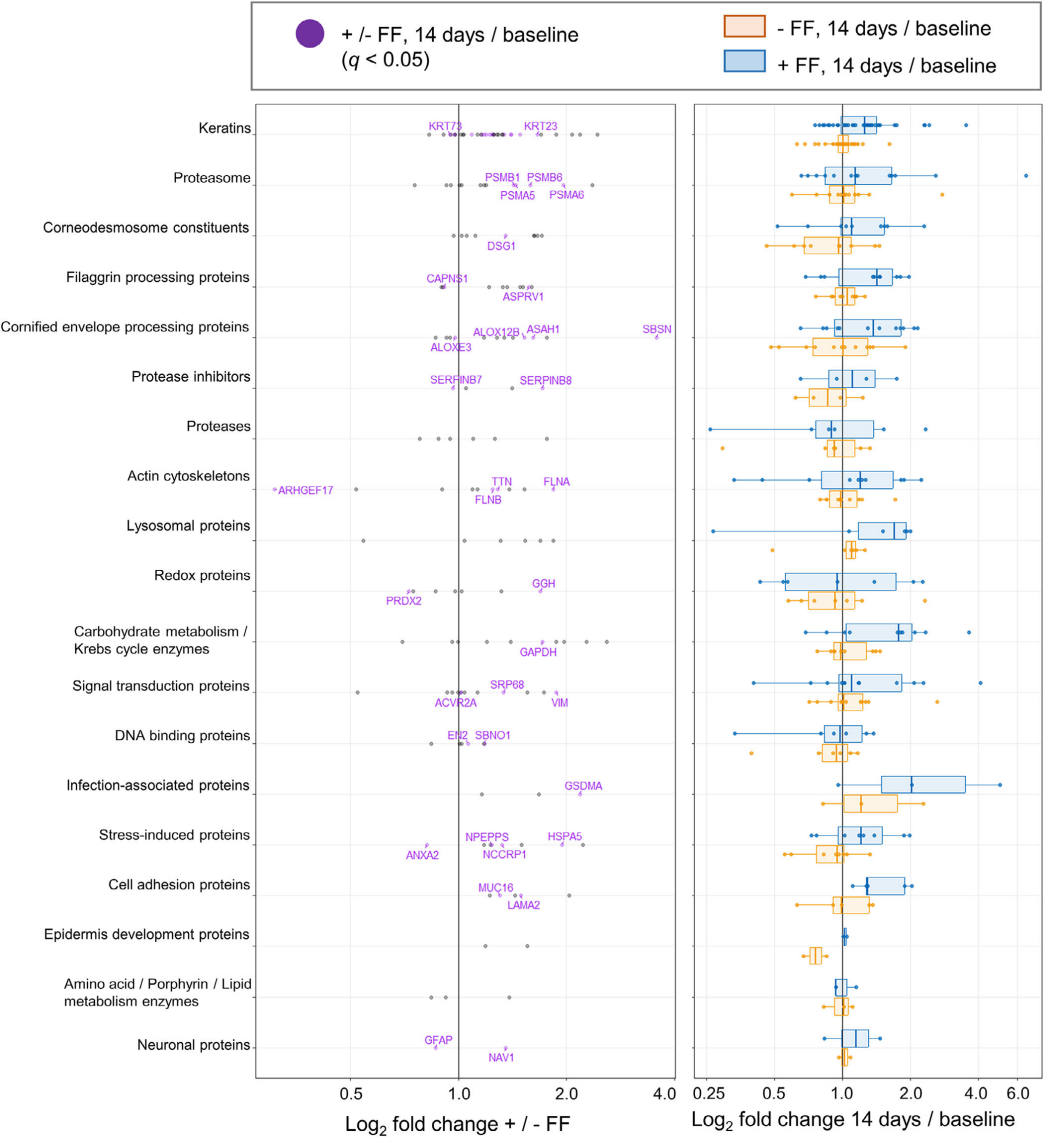
Changes in the SC proteome after 14 days by functional group. Dot plots showing log_2_ fold change between with and without FF (marked proteins, significant increase or decrease). Box plots showing the log_2_ fold change after treatment (orange: without FF, blue: with FF).

A total of 172 proteins were identified in the SC samples collected by the tape strip method. First, the difference in the protein levels between the lotion alone group and with the FF film group at baseline was calculated from PAI. The 163 proteins with absolute differences between these groups at baseline within 95% of the total were selected for analysis, and nine proteins with large differences at baseline (Hemoglobin subunit beta (3.8×), Protein S100‐A7 (3.2×), Protein S100‐A9 (3.1×), Heat shock cognate 71 kDa protein (2.8×), Tuftelin (2.5×), Calmodulin‐like protein 5 (2.4×), Interleukin‐37 (2.1×), Plasminogen activator inhibitor 2 (1.9×), and Phosphoglycerate kinase 1 (1.9×)) were excluded from the analysis. Next, the fold change from baseline to day 14 was calculated for the 163 proteins. Proteins increased by both lotion alone and in combination with the FF film are shown in the upper‐right quadrant (significant differences in 29 of 76 proteins), and those decreased by both lotion alone and in combination with the FF film are shown in the lower‐left quadrant (significant differences in 9 of 50 proteins) in Figure 2. The proteins that increased with the FF film but decreased with lotion alone are shown in the upper‐left quadrant (significant differences in 10 of 33 proteins), and those that increased with lotion alone but decreased with the FF film are shown in the lower‐right quadrant (significant difference in 1 of 4 proteins). Many proteins showed similar changes with lotion alone and in combination with the FF film, indicating positive correlations. Furthermore, the ratios of the fold change from baseline to Day 14 were calculated between with and without the FF film and are shown in Table S3. When categorized by protein function, the data indicate that, for many functional groups, the associated proteins were increased by the FF film combination (Figure 3). Keratins 1, 2, 3, 5, 7, 19, 23, 26, 27, 28, 75, and 80 increased more with the FF film than with lotion alone. In contrast, keratins 8, 15, and 73 decreased more with the FF film than with lotion alone. Since keratin 14 is expressed in the basal cell layer together with keratin 5, its increase suggests hyperproliferation [16], but there was no significant increase in keratin 14. Keratins 6, 16, and 17 are similar keratins whose increase suggests overgrowth [17], but no significant increase was observed. Proteasome subunit alpha type 5 (PSMA5) and 6 (PSMA6), and proteasome subunit beta type 1 (PSMB1) and 6 (PSMB6) were increased with the FF film. Of the corneodesmosome constituents, desmoglein‐1 was increased with the FF film. For filaggrin processing proteins, retroviral‐like aspartic protease 1 (ASPRV1), which hydrolyzes filaggrin polymer (poly‐FLG) to filaggrin monomer (mono‐FLG) [18, 19], was increased with the FF film, whereas calpain small subunit 1 (CAPNS1), an enzyme that further hydrolyzes mono‐FLG, decreased. Although mono‐FLG hydrolytic enzymes such as bleomycin hydrolase (BLMH) and caspase‐14 (CASP14), and amino acid synthases such as arginase‐1 (ARG1) and histidine ammonia‐lyase (HAL) were also identified, there were no significant differences between the lotion alone and the combination with the FF film. For cornified envelope (CE) processing proteins, suprabasin (SBSN), which is involved in the formation of the skin barrier [20, 21], acid ceramidase (ASAH1), which is involved in the maintenance of skin ceramide homeostasis [22], and arachidonate 12‐lipoxygenase, 12R‐type (ALOX12B), which catalyzes the formation of bound ceramide [23], were significantly increased by the combination with the FF film. Hydroperoxide isomerase ALOXE3 (ALOXE3) is involved in the formation of bound ceramide together with ALOX12B, but it was very slightly reduced by the combination with the FF film. Serpin B8 (SERPINB8), which is low in exfoliative ichthyosis [24], was increased by the combination with the FF film. As for the cytoskeleton, the FF film combination increased filamin‐A (FLNA), filamin‐B (FLNB) [25], and titin (TTN) and decreased rho guanine nucleotide exchange factor 17 (ARHGEF17). Of the redox proteins, the combination with the FF film increased gamma‐glutamyl hydrolase (GGH), which regulates cysteine levels in the body [26], and decreased peroxiredoxin‐2 (PRDX2), which is elevated in psoriasis [27]. Glyceraldehyde‐3‐phosphate dehydrogenase (GAPDH), a response enzyme of energy metabolism, vimentin (VIM), which is increased in redifferentiation after wound healing [28], signal recognition particle subunit SRP68 (SRP68), and activin receptor type‐2A (ACVR2A) were increased by the combination with the FF film. Gasdermin‐A (GSDMA), a marker of improved skin barrier function [29], endoplasmic reticulum chaperone BiP (HSPA5), associated with misfolded quality control [30], and puromycin‐sensitive aminopeptidase (NPEPPS) [31] were increased by the combination with the FF film, and annexin A2 (ANXA2) [32], a dry skin marker, was decreased. Laminin subunit alpha‐2 (LAMA2), a basement membrane component that decreases with age, was increased by the combination with the FF film. Other proteins that were examined, but whose relationship with skin function is not clear, were protein strawberry notch homolog 1 (SBNO1), homeobox protein engrailed‐2 (EN2), F‐box only protein 50 (NCCRP1), mucin‐16 (MUC16), and neuron navigator 1 (NAV1), and they were increased by the combination with the FF film, whereas glial fibrillary acidic protein (GFAP) was decreased.

### 3.2 Clinical Study 2

**FIGURE 4:**
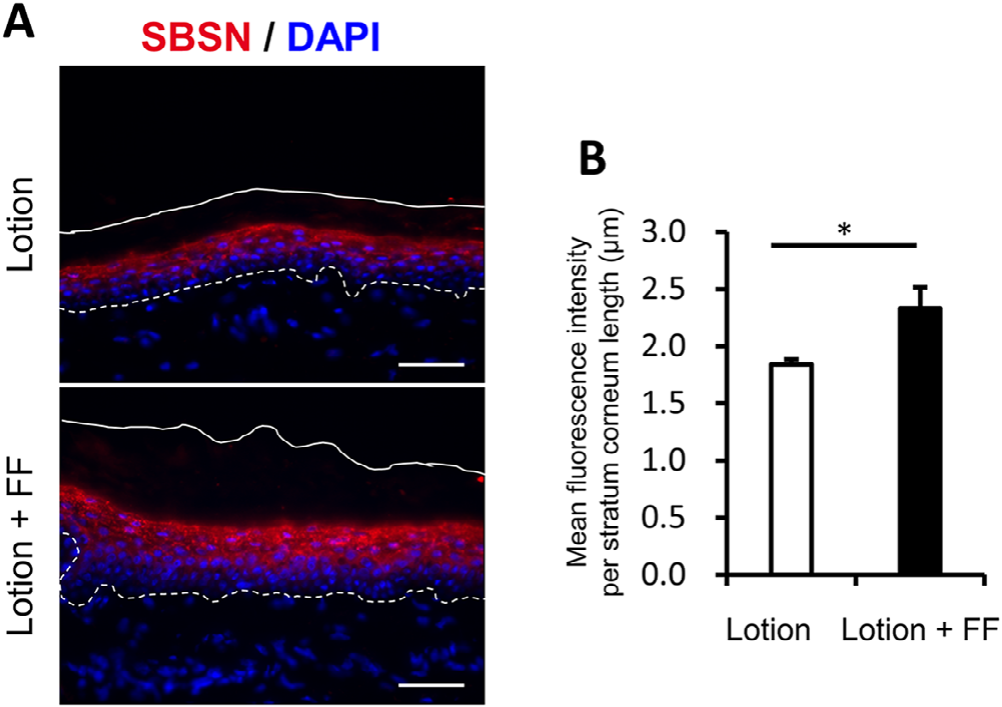
Characterization of SBSN fluorescence intensity in the epidermis. (A) Immunofluorescence staining for SBSN (red). Skin tissues were procured 5 days after two types of treatments (lotion alone and lotion with the FF film). The top solid line indicates the skin surface. The bottom dotted line indicates the boundary between the epidermis and the dermis. Bar = 50 µm. (B) The mean SBSN fluorescence intensity per SC length in lotion and lotion with FF‐treated skin. Values are shown as means ± SD (*n* = 3) (*p < 0.05 by paired *t*‐test).

**FIGURE 5:**
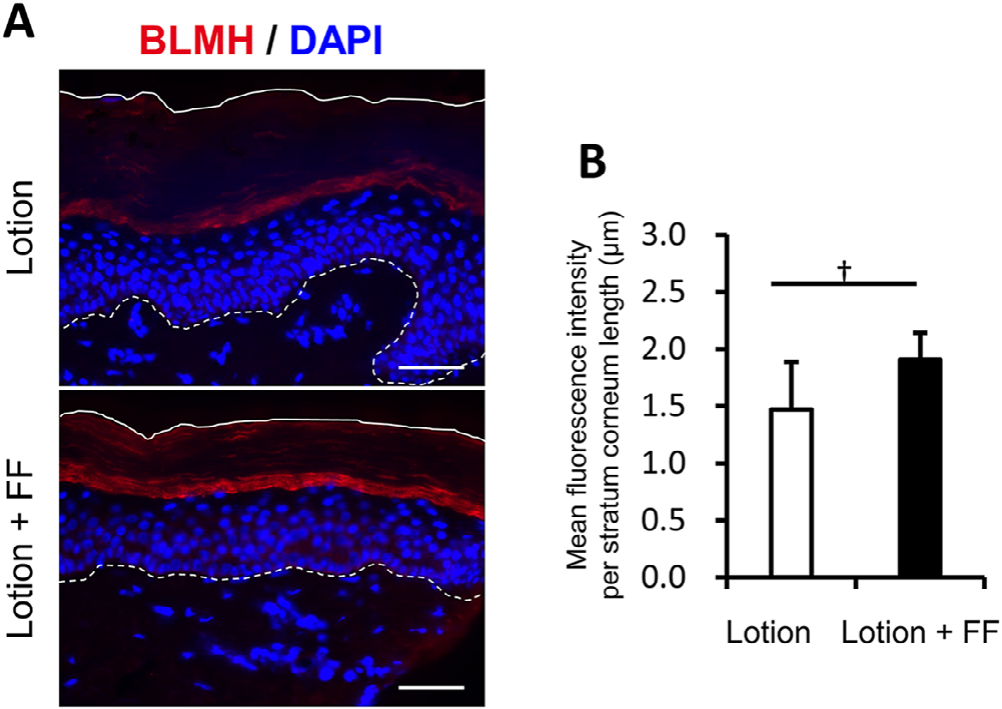
Characterization of BLMH fluorescence intensity in the epidermis. (A) Immunofluorescence staining for BLMH (red). Skin tissues were procured 3 days after two types of treatments (lotion alone and lotion with the FF film). The top solid line indicates the skin surface. The bottom dotted line indicates the boundary between the epidermis and the dermis. Bar = 50 µm. (B) The mean BLMH fluorescence intensity per SC length in lotion and lotion with FF‐treated skin. Values are shown as means ± SD (*n* = 4) (†*p* < 0.1 by paired *t*‐test).

In the previous study, skin changes were observed in the first week and continued to improve thereafter, and changes were also observed in the SC proteome after 2 weeks. Skin biopsies were performed 3 or 5 days after the combination of lotion and the FF film to confirm the changes within the skin. Seven healthy female subjects with dry skin on the lower legs (initial observer dryness score ≥ 2.5) were recruited and tested using lotion alone and lotion with the FF film under the same conditions as before. Both lotion alone and with the FF film improved observer scores and increased SC water content (conductance) at 3 and 5 days, with a significant increase in conductance at 5 days with the FF film compared with lotion alone (Figure S2). Immunohistochemical staining was performed for BLMH, ASPRV1, SBSN, ALOX12B, and desmocollin‐1 (DSC‐1), which were largely changed on SC proteome analysis as putative factors directly related to the improvement of the skin condition. In the comparison between lotion alone and in combination with the FF film, the fluorescent signal tended to be higher for SBSN, which is related to skin barrier function, and for BLMH, which is related to water retention function (Figures S3 and S4). For SBSN, a representative immunostaining image was shown in Figure 4A, and a significant increase in the fluorescence signal was observed when the combination with the FF film was used for 5 days compared with lotion alone (Figure 4B). For BLMH, a representative immunostaining image was shown in Figure 5A, and an increasing trend in the fluorescence signal was also observed when the FF film was used in combination with lotion for 3 days compared with lotion alone (Figure 5B). However, no significant changes were observed in ASPRV1, ALOX12B, and DSC‐1 (data not shown). Thus, even over the short period of 3–5 days, the combination of lotion and the FF film induced significant changes in the expressions of proteins involved in the maintenance of epidermal homeostasis within the skin.

## 4 Discussion

In clinical studies 1 and 2, both observer scores and conductance improved earlier with the FF film combination. Protein expression levels in skin tape strips after 14 days of the FF film use and skin biopsies after several days (3 or 5 days) of the FF film use were compared with those of lotion alone to investigate the biological changes in the skin induced by the FF film.

First, when examining the overall SC proteome, the correlation coefficient (r) for the scatter plot in Figure 2 was 0.775, suggesting that the SC proteome largely reflected the action of the lotion. In addition, the combination with the FF film increased many proteins more than lotion alone (fold‐change >1, Figure 3). Moreover, the FF film combination was found to decrease ANXA2, high in dry skin [32], and PRDX2, high in psoriasis [27], and increase SERPINB8, low in exfoliative ichthyosis [24], and GSDMA, low in atopic dermatitis (AD) [29]. The data suggest that the improvement of dry skin symptoms with the combination of the FF film is accompanied by improvements in the abnormalities of the biological mechanisms observed in the above skin diseases. The increased expression of keratin 5, which is originally highly expressed in the basal layer, and FLNA and FLNB, which are highly expressed in the supra‐basal layer, observed in combination with the FF film, is one of the factors suggesting epidermal overgrowth. However, the same basal keratin, keratin 14, and supra‐basal keratins, keratin 6, keratin 16, and keratin 17, did not show significant increases, suggesting that they do not correspond to the enhanced cell proliferation. Therefore, the findings were not considered to correspond to enhanced cell proliferation that is observed in sun‐exposed areas compared with unexposed areas, for example. However, many keratins, including their subtypes that showed no significant differences between the lotion alone and the combination with the FF film, tended to increase with the FF film (Figure 3), suggesting that epidermal differentiation rather than overproliferation was induced. SBSN, also known as lamellar body (LB) secreted protein, has been reported to be involved in epidermal differentiation and healthy barrier function [33]. SBSN changed 0.5‐ and 1.9‐fold from baseline when lotion was used alone and with the FF film, respectively, which also suggests that the FF film promotes epidermal cell differentiation. VIM, a protein known to regulate fibroblast proliferation and keratinocyte differentiation via TGF‐β‐Slug signaling during wound healing [28], increased 1.2‐ and 2.3‐fold from baseline when lotion was used alone and with the FF film, respectively; these data also suggest that the FF film promotes differentiation. Taken together, these results suggest that the FF film induced mild cell proliferation and epidermal differentiation without leading to overproliferation. In this study, loricrin, involucrin and small proline rich proteins (SPRs), which are major precursor proteins of the CE, and differentiation‐induced proteins were not detected. One possible reason is that these proteins are cross‐linked by isopeptide bonds. Consequently, they were not cleaved under the present enzymatic treatment conditions, or the theoretical mass calculated from the amino acid sequence in the database did not match the measured value. In the previous study [34], SC proteome analysis of AD showed that involucrin was detected in AD patients, but not in healthy subjects. This result suggests that CEs in healthy skin are in a more cross‐linked mature state than in AD patients, resulting in them being difficult to detect by proteome analysis. In another report [35], loricrin, involucrin, or SPRs were detected in neither AD patients nor healthy subjects. In the former case, 8 consecutive D‐Squame tapes at the antecubital fossae site and a cellophane tape at the forearm in the latter case were used to collect SC samples. Thus, the collection method and site may affect the protein detection in addition to skin condition. Further study is necessary to investigate the influence of the FF film on CE precursors. In addition, the FF film treatment increased proteasome‐related proteins, especially PSMB6, which is essential for the 20S proteasome subunit, and HSPA5 [30] and NPEPPS [31], which are involved in the regulation of protein misfolding. These results suggest that the quality control mechanism of protein metabolism may have been normalized by the FF film treatment. Furthermore, ASAH1, an enzyme that regulates the metabolism of ceramide [22], which plays an important role in skin barrier function, and ALOX12B [23], an enzyme that catalyzes the formation of the cornified lipid envelope (CLE), a complex of ceramide and keratinocyte proteins, were both increased with the FF film. These findings suggest normalization of ceramide metabolism. ASAH1 degrades ceramide into sphingosine and fatty acids, and the degraded sphingosine is also reused as a substrate for ceramide. Since ceramide is one of the key factors for skin barrier function, the normalization of this mechanism was thought to contribute to the improvement of dry skin. Moreover, sphingosine 1‐phosphate, a phosphorylated form of sphingosine, functions as an intracellular signal transduction molecule and regulates the production of cathelicidin antimicrobial peptide (CAMP) [36], and the combination of lotion and the FF film may normalize the physical and chemical barrier mechanisms of the skin. Since no ceramide‐metabolizing enzymes other than ASAH1 were detected in this proteome analysis, the changes that occurred in ceramide metabolism remained unclear. Therefore, it will be necessary to elucidate the effect of the FF film on ceramide metabolism using, for example, 3D‐reconstructed skin in the future. As mentioned above, SBSN is a secreted protein encapsulated in LB, but since LB is a secretory cell organelle that contains various enzymes, ceramides, and antimicrobial peptides, the promotion of LB secretion contributes to the maintenance of skin homeostasis [37]. In the present study, LB secretion was not examined, but immunohistochemical staining of skin biopsies showed an increase in SBSN expression after 5 days of the FF film use. Therefore, the combination of lotion and the FF film may have promoted LB secretion, supplied various keratinization‐related factors contained in LB to the epidermis, and restored the entire keratinization. For example, the FF film combination was found to increase expressions of ASPRV1, an LB‐secreted protein, and SBSN. ASPRV1 is an enzyme that cleaves profilaggrin, a series of mono‐FLG, as they migrate from the granular layer to the SC. This reaction is an essential process to produce natural moisturizing factor (NMF), and since lotion alone and in combination with the FF film increased ASPRV1 1.3‐ and 2.0‐fold from baseline, respectively, it was expected that filaggrin levels would increase, but no significant increase was observed in the SC proteome analysis. Even though ASPRV1 expression in the immunohistochemical examination of skin biopsies was unchanged, this could be attributed to the fact that ASPRV1 expression may require slightly more time to increase than SBSN. Regarding other enzymes that hydrolyze filaggrin, little change was observed in CASP14, whereas BLMH and GGCT, which convert filaggrin‐hydrolyzed amino acids to pyrrolidone carboxylic acid (PCA), and HAL, which converts filaggrin‐hydrolyzed amino acids into urocanic acid (UCA), were increased by concomitant use of the FF film, but no significant difference was observed. However, a trend toward increased expression of BLMH was observed on immunohistochemistry of skin biopsies 3 days after the combination, suggesting that BLMH may be involved in the clinical improvement of SC water content with the FF films.

The relationship between biopsy samples taken 3 and 5 days after use of the formulation and SC samples taken 14 days later also needs to be considered. Immunohistochemical examination of skin biopsies showed that SBSN and BLMH expressions were promoted in the granular layer within 5 days of the FF film combination use. In skin tape strippings collected 14 days after the FF film use, SBSN also showed the largest increase (3.6‐fold), with a nonsignificant 1.6‐fold increase in BLMH. The fact that the combined use of the FF film promoted improvement in SC water content even over a short period of 5 days suggests that SBSN and BLMH may have provoked initial changes, although no significant changes in other factors related to water content and adhesion were detected with the combined use of the FF film.

The turnover of the epidermis and SC is known to be about 39 days [38] and 14 days, respectively. Although the effect of the FF film on turnover of the SC or epidermis is unclear, assuming no change in turnover rate, it was reasonably assumed that changes that occurred near the upper layer of the granular layer within a few days of treatment would have appeared in the tape stripping samples of SC 14 days later. Thus, the changes observed in the proteome analysis of skin tape strippings in the present study were presumably due to the action of the FF film. The proteome changes were also linked to improvements in skin condition indices such as observer scores and conductance, suggesting that the FF film has a strong effect to normalize the keratinization process of dry skin, resulting in earlier skin improvement than lotion alone.

The results of the present study were based on a limited number of samples, and additional investigation is needed to elucidate influences of the FF film on the skin including the time lag between the epidermis and the SC. In particular, effects of the FF film on changes in the constituent molecules of corneodesmosomes and CE, which are assumed to contribute to the observer score, and on molecules associated with the process of NMF production from filaggrin, which is assumed to contribute to the conductance value were not fully demonstrated. We believe that a re‐examination with a larger number of subjects and an extended treatment period is necessary to examine in detail the effects of the FF film on the skin and the relationship between the biopsy samples and the SC samples.

## 5 Conclusion

In this study, skin biopsies after several days and skin tape stripping proteins after 14 days of continuous use were analyzed to investigate the biological responses in the skin that contribute to the early improvement of dry skin observed when lotion and the FF film are used together. The FF film increased the expression of SBSN near the granular layer within a few days, and this change could be detected by skin tape stripping after 14 days. A detailed study of the proteome contained in the skin tape stripping suggested that the combination of the FF film and lotion induced mild cell proliferation and promoted epidermal differentiation, protein and ceramide metabolism, and LB secretion. Although further study is needed, the present findings provide new insights not only into the treatment of dry skin, but also into skin diseases such as psoriasis and AD.

## Conflicts of Interest

The authors declare that there is no conflict of interest that could be perceived as prejudicing the impartiality of the research reported. The authors are employees of Kao Corporation and Kao USA Inc.

## Ethics Statement

The tape stripping study was conducted according to the principles of the Declaration of Helsinki using protocols ethically reviewed and approved by the organization Kao USA Inc. The biopsy studies performed in the United States were approved by Schulman Associates Institutional Review Board, Inc. (Cincinnati, OH, USA) (protocol numbers 17‐124‐NCC/20‐1703, 18‐009‐NCC). Written informed consent to participate in all studies was obtained from each subject before the start of the study.

## Supporting information



Supporting Information

Supporting Information

Supporting Information

Supporting Information

Supporting Information

Supporting Information

## Data Availability

The data that support the findings of this study are available from the corresponding author upon reasonable request.
