# Supplementary material for: Biological Effects of a Fine Fiber Film Treated With a Lotion to Improve Dry Skin
Source: Skin Res Technol. 2025 May 5;31(2-5):e70161. doi: 10.1111/srt.70161 (PMC12050644; doi:10.1111/srt.70161)
Supplement: Supplementary file 1 — Supporting Information [file SRT-31-e70161-s004.docx]

TABLE S1. Lotion composition

| **Ingredient** | **Content (% wt/wt)** |
| --- | --- |
| Petrolatum White | 75.0 |
| Cetyl Alcohol | 1.8 |
| Stearyl Alcohol | 1.2 |
| Glycerin | 3.0 |
| Phenoxyethanol | 0.3 |
| PEG-60 Hydrogenated Castor Oil | 0.4 |
| Butylene Glycol | 1.0 |
| Water | 17.3 |

TABLE S2. Fiber composition for electro-spinning

| **Ingredient** | **Content (% wt/wt)** |
| --- | --- |
| Poly vinyl butyral | 11.0 |
| Phytosteryl/Octyldodecyl Lauroyl Glutamate | 4.6 |
| Ethanol | 84.0 |
| Water | 0.4 |
