# Supplementary figures and images for: Biological Effects of a Fine Fiber Film Treated With a Lotion to Improve Dry Skin
Source: Skin Res Technol. 2025 May 5;31(2-5):e70161. doi: 10.1111/srt.70161 (PMC12050644; doi:10.1111/srt.70161)

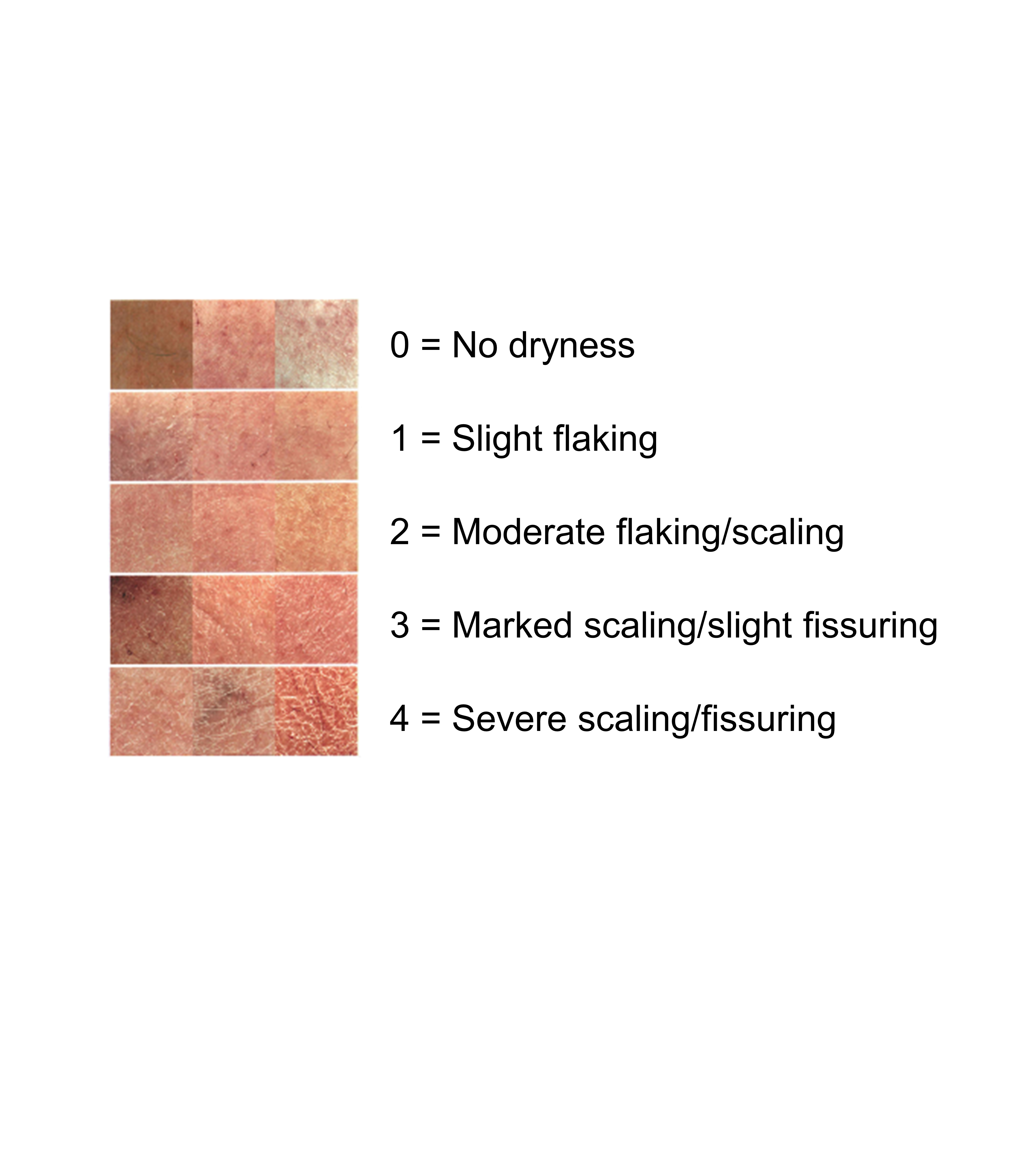

Supplement: Supplementary file 3 — Supporting Information [file SRT-31-e70161-s002.TIF]

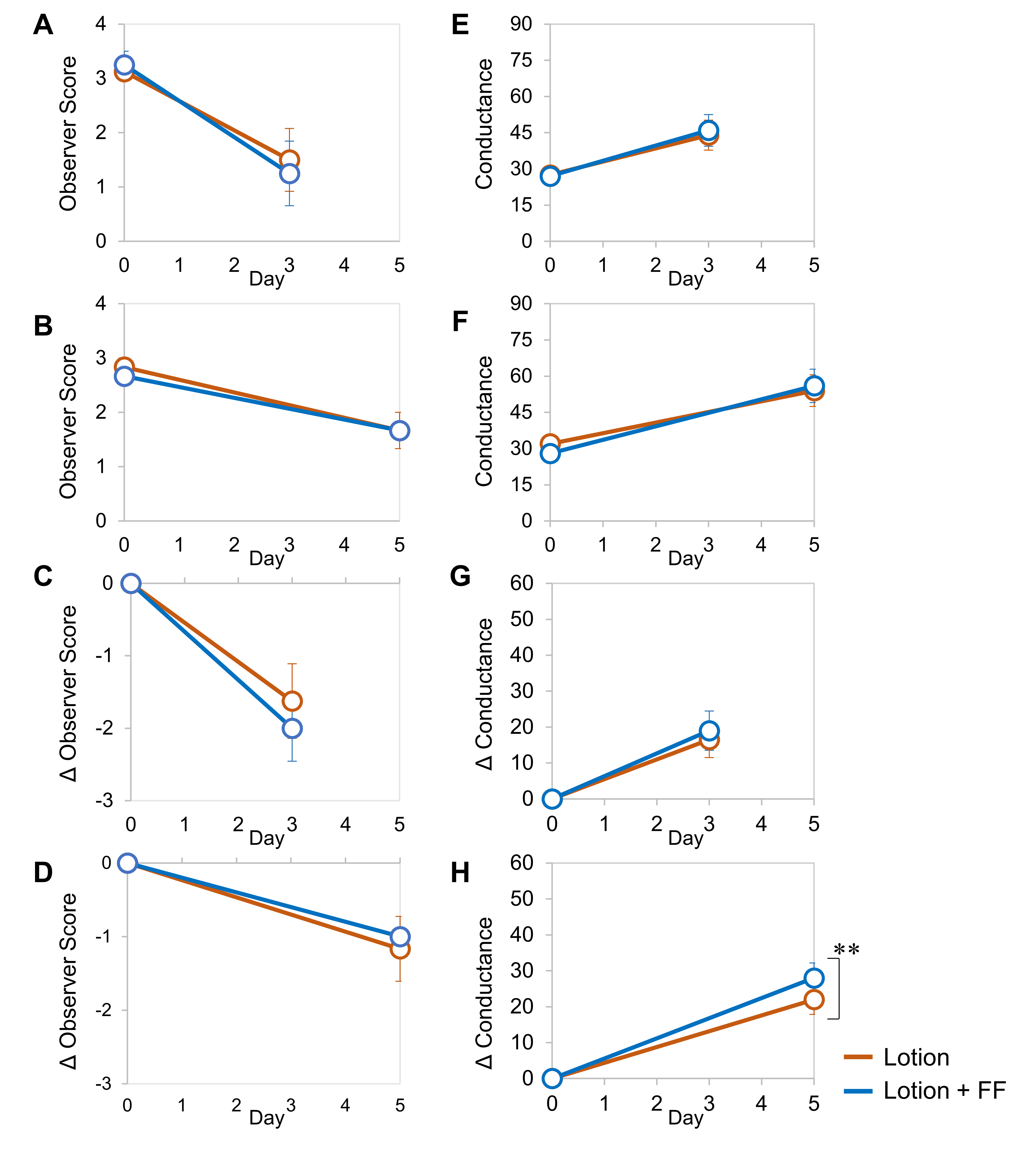

Supplement: Supplementary file 4 — Supporting Information [file SRT-31-e70161-s003.TIF]

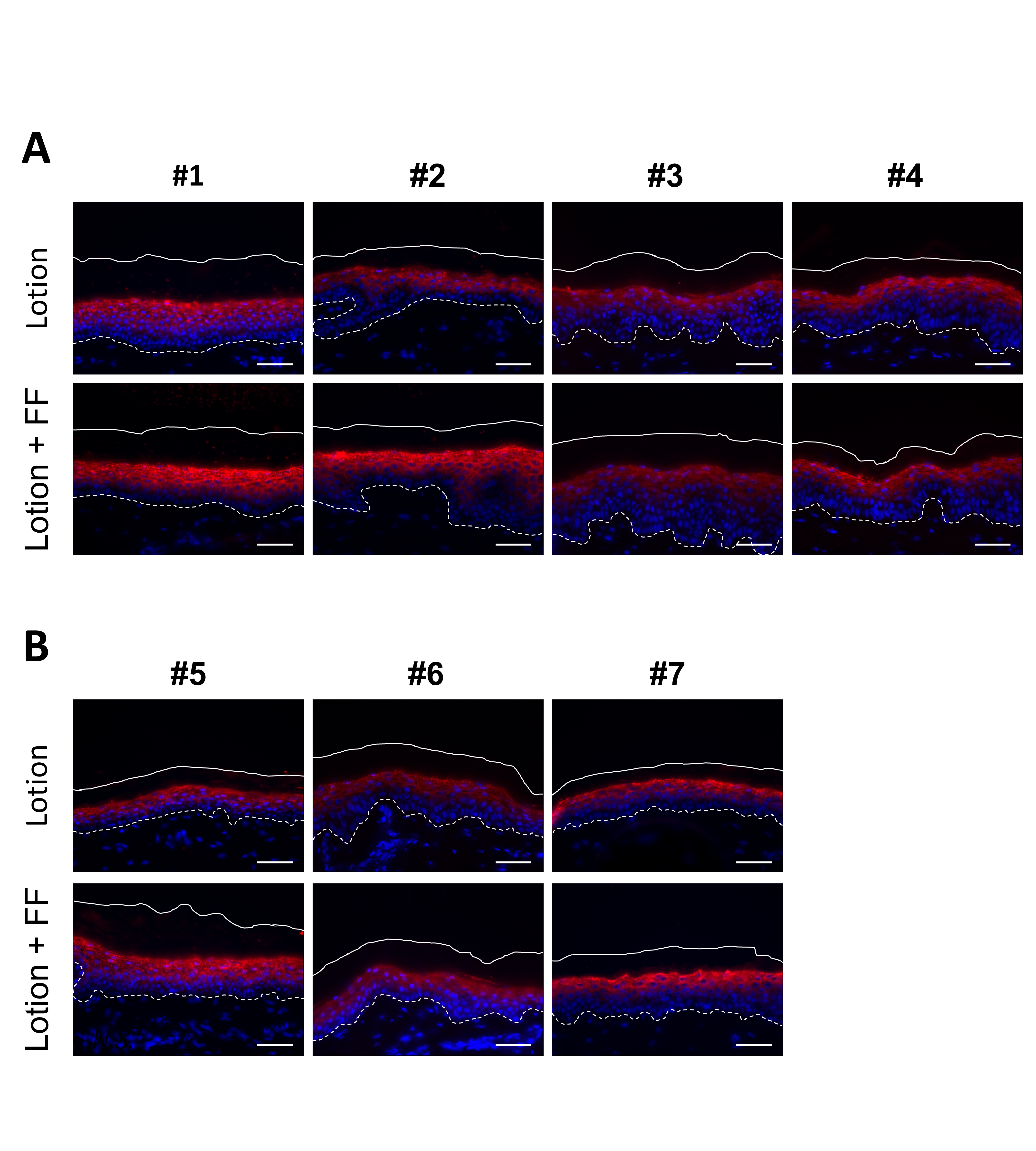

Supplement: Supplementary file 5 — Supporting Information [file SRT-31-e70161-s005.TIF]

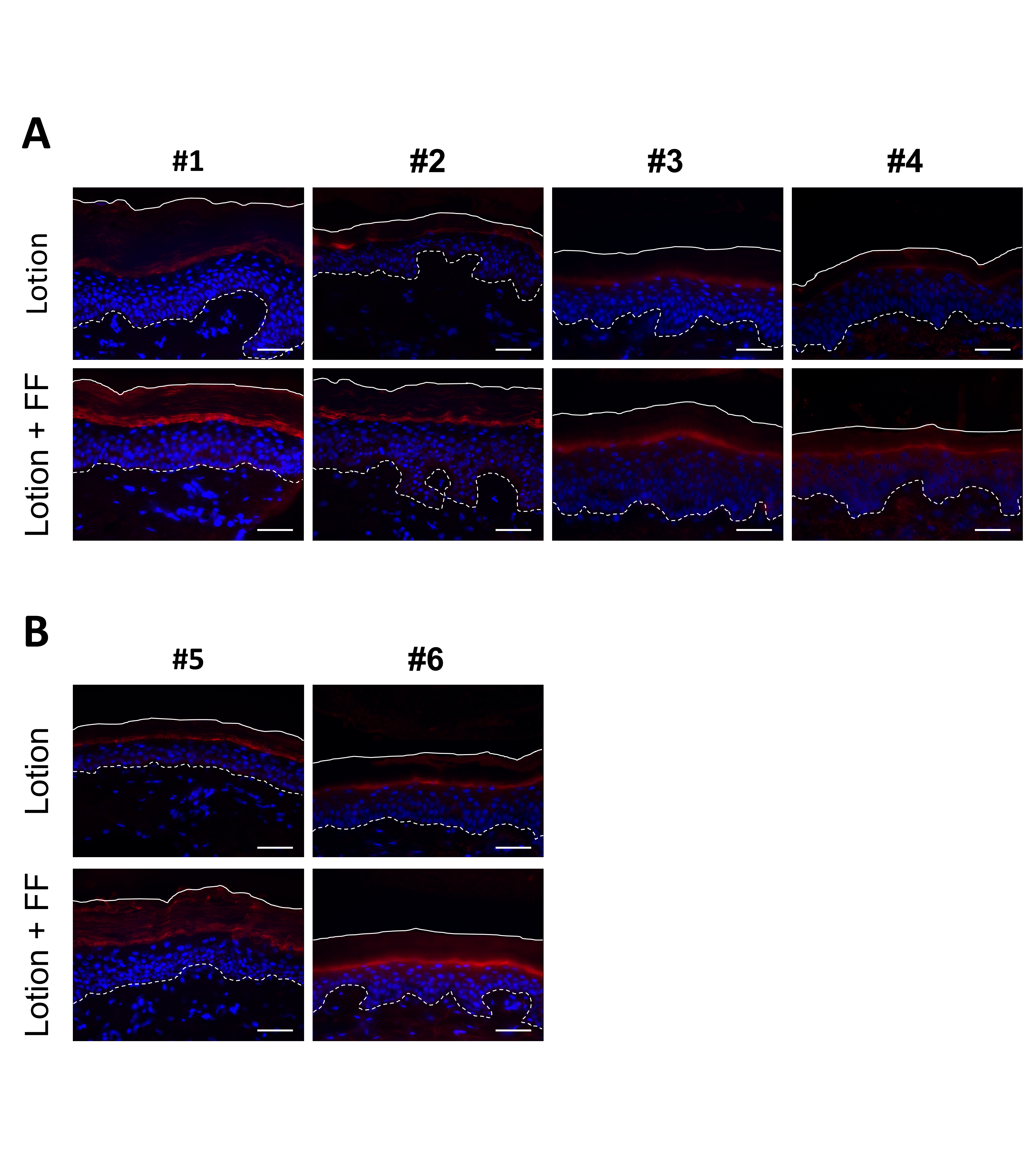

Supplement: Supplementary file 6 — Supporting Information [file SRT-31-e70161-s001.TIF]
